# National geographical pattern of COVID-19 hospitalization, case fatalities, and associated factors in patients covered by Iran Health Insurance Organization

**DOI:** 10.1186/s12889-022-13649-0

**Published:** 2022-06-30

**Authors:** Soheila Damiri, Ali Shojaee, Mohsen Dehghani, Zahra Shahali, Sadrollah Abbasi, Rajabali Daroudi

**Affiliations:** 1 Department of Health Management and Economics, School of Public Health, Tehran University of Medical Sciences, Poursina Ave., Tehran, 1417613191 Iran; 2 National Center for Health Insurance Research, Tehran, Iran; 3 Department of Epidemiology, School of Public Health, Iran University of Medical Sciences, Tehran, Iran

**Keywords:** COVID-19, Case Fatality Rate, Mortality, Health Status Disparities, Hospitalization, Intensive Care Units, Geographic Locations, Iran

## Abstract

**Background:**

Understanding the Spatio-temporal distribution and interpersonal comparisons are important tools in etiological studies. This study was conducted to investigate the temporal and geographical distribution of COVID-19 hospitalized patients in the Iran Health Insurance Organization (IHIO) insured population (the second largest social health insurance organization) and the factors affecting their case fatality rate (CFR).

**Methods:**

In this descriptive-analytical cross-sectional study, the demographic and clinical data of all insured of the IHIO who were hospitalized with COVID-19 in hospitals across the country until March 2021 was extracted from the comprehensive system of handling the inpatient documents of this organization. The Excel 2019 and GeoDA software were used for descriptive reporting and geographical distribution of variables. A multiple logistic regression model was used to estimate the Odds Ratio (OR) of death in patients with COVID-19 using STATA 14 software.

**Results:**

During the first 14 months of the COVID-19 outbreak in Iran, 0.72% of the IHIO insured (303,887 individuals) were hospitalized with COVID-19. Hospitalization per 100,000 people varied from 192.51 in East Azerbaijan to 1,277.49 in Yazd province. The overall CFR in hospitalized patients was 14%. Tehran and Kohgiluyeh & BoyerAhmad provinces had the highest and lowest CFR with 19.39% and 5.19%, respectively. The highest odds of death were in those over 80 years old people (OR = 9.65), ICU-admitted (OR = 7.49), Hospitalized in governmental hospitals (OR = 2.08), Being a foreign national (OR = 1.45), hospitalized in November (OR = 1.47) and Residence in provinces such as Sistan & Baluchestan (OR = 1.47) and Razavi Khorasan (OR = 1.66) respectively. Furthermore, the odds of death were lower in females (OR = 0.81) than in males.

**Conclusions:**

A sound understanding of the primary causes of COVID-19 death and severity in different groups can be the basis for developing programs focused on more vulnerable groups in order to manage the crisis more effectively and benefit from resources more efficiently.

**Supplementary Information:**

The online version contains supplementary material available at 10.1186/s12889-022-13649-0.

## Background

The COVID-19 pandemic has been the greatest challenge facing humanity since the 1918 flu pandemic [1]. Until February 23, 2022, 426,624,859 people have been reported to the World Health Organization (WHO) to be infected with this disease, of which 5,899,579 died [2]. These cases are official statistics reported, but according to some estimates, the number of fatalities due to COVID-19 has been around 11 million so far [3]. The COVID-19 crisis has threatened development achievements for decades [4].

As communities, health organizations, and governments continue to fight the COVID-19 pandemic, understanding how its impact changes the space and among population groups is crucial [5]. “Space” can usually be used as an alternative to the interaction between genetic factors, lifestyle, and environment. Although the role of space in human health has been historically recognized, the focus in public health research has been basically on person and time, and spatial aspects have received less attention [6]. The current COVID-19 outbreak has led to a re-understanding of new health risks and vulnerabilities. Neglected vulnerabilities may be due to the socio-biological combination of local communities. Despite the fact that, in micro-epidemics, the local maps of the disease have been used to track yellow fever cases in New York during 1796 and 1797. Prior to the COVID-19 outbreak, only a few studies had examined the distribution of health risks and vulnerabilities among communities [7]. Considering the space dimension along with temporal and interpersonal comparisons can be a helpful tool for developing and testing etiological hypotheses. In addition, from a pragmatic perspective on public health, awareness of the focus of a health problem in identifiable locations is essential for the efficient distribution of resources for prevention, treatment, or improvement [6]. Local population dynamics and socio-demographic characteristics have been proven to affect the transmissibility and prevalence of COVID-19 disease, but initial control measures have not taken into account location-based differences in exposure and consequences [8]. Nevertheless, after the COVID-19 pandemic, numerous researchers around the world, including the United States [9–11], Italy [12], the United Kingdom [8, 13], Denmark [14], Sweden [15], Brazil [16], etc. have tried to investigate the spatial-geographical dimensions of the pandemic distribution.

On February 18, 2020, the Iranian government officially confirmed identifying the first cases of COVID-19 [17, 18]. Until February 23, 2022, the number of confirmed cases of infection in Iran was 6,983,635, among which 135,499 have died; 1.94% of all patients and 2.29% of world deaths have occurred in Iran [2]. Studies conducted in recent years have provided considerable evidence of mild to severe degrees of geographical differences in social determinants of health, healthcare infrastructure and services [19–21], and health consequences [22, 23]. During the COVID-19 pandemic, several studies have also evaluated the spatial and geographical distribution of the pandemic in Iran [24–29]. The COVID-19 pandemic has put pressure on hospital systems worldwide with the rapid and long-term increase in the flow of patients to hospitals. Hospital management strategies and patient consequences vary internationally and are a function of infection rate, public health structures, and organizing the healthcare system. In addition, analyzing COVID-19-induced fatality in several countries shows differences in patient consequences across different regions, in part due to demographic differences in population. However, limited analyses of inter-hospital variations in COVID-19 case fatality rates (CFRs) have been published [30]. The above studies conducted to investigate the spatial dimensions of the pandemic in Iran, despite creating an understanding of the spatial/temporal distribution of COVID-19 at the national and local levels, have not been based on hospital macro data. The present study was conducted to provide evidence of inter-hospital consequences of patients with COVID-19 among the population insured by Iran Health Insurance Organization (IHIO), an organization with about 42 million insured individuals in 2019 [31] so that it represents almost half of the country’s population.

## Methods

### Study design

In this descriptive-analytical cross-sectional study, the geographical distribution of hospitalization cases and case fatalities due to COVID-19 in the population insured by IHIO was investigated. Iran Health Insurance Organization is a government organization affiliated with the Ministry of Health and Medical Education (MoHME) in Iran [32]. This organization is the largest public sector insurance organization in Iran after the Social Security Organization, which had about 42 million insured in 2019 [31]. For a better understanding of the geographical distribution of the insured population of this organization and the distribution of hospital beds in the provinces of Iran, see Table A1 in Additional file 1. All missions of IHIO are focused on health and some of them are: Expand health insurance coverage, Achieve equitable coverage of health services, Achieve universal health coverage [33]. The organization has five main funds, which are:

1. The Fund of the Civil Servants: All civil servants including the employed, the retired, the pensioner and the employee whose retirement deductions are paid to the Civil Servants Pension Organization covered by this fund.
2. Rural Fund: The insured people of this fund include all villagers, nomads and the residents of cities with less than 20,000 people
3. Iranian & Universal Health Insurance Funds: All Iranian applying for insurance can be covered by Iranian insurance or universal health insurance via full premium payment or payment based on the household income level.
4. Foreign Citizens Fund: This fund is for non-Iranian nationals.
5. The Other Social Strata: The other social groups which are not the beneficiaries of above-mentioned funds are covered under this fund such as Veterans including martyrs’ family, warfare victims and released captives of war, students, Disabled and clients covered by the State Welfare Organization, Clients covered by Imam Khomeini Relief Committee, Prisoners and their families, and etc.

**Table 1:** Factors related to the death of patients hospitalized due to COVID-19 in the population insured by Iran Health Insurance Organization until March 20, 2021 (based on the multiple logistic regression model)

Variable		Survived		Dead		Odds Ratio			*P*-Value
**Variable**	**Sub groups**	**Frequency**	**%**	**Frequency**	**%**	**OR**	**95% CI**^a^		
Insurance fund	Iranian Fund	18,070	15.43	3,298	15.43	Ref			
	Rural Fund	100,405	87.81	13,943	12.19	0.75	0.72	0.79	*P* < 0.001
	Other Social Strata	33,076	89.95	6,800	17.05	0.92	0.87	0.98	*P* < 0.001
	Foreign Citizens Fund	1,445	77.44	421	22.56	1.45	1.28	1.66	*P* < 0.001
	Universal health insurance	45,853	88.08	6,206	11.92	0.84	0.81	0.89	*P* < 0.001
	Fund of the Civil Servants	62,328	83.99	11,880	16.01	0.82	0.79	0.87	*P* < 0.001
Age group	< 20	14,111	95.25	703	4.75	Ref			
	20–30	13,092	95.88	562	4.12	1.15	1.03	1.3	*P* < 0.001
	30–40	26,073	94.94	1,389	5.06	1.45	1.32	1.6	*P* < 0.001
	40–50	32,335	92.93	2,459	7.07	2.09	1.92	2.3	*P* < 0.001
	50–60	44,954	90.18	4,895	9.82	2.89	2.66	3.15	*P* < 0.001
	60–70	54,362	85.48	9,232	14.52	4.39	4.05	4.78	*P* < 0.001
	70–80	41,066	79.97	10,287	20.03	6.48	5.96	7.03	*P* < 0.001
	> 80	35,327	73.04	13,040	26.96	9.65	8.89	10.49	*P* < 0.001
Gender	Male	131,635	84.64	23,883	15.36	Ref			
	Female	129,685	87.41	18,684	12.59	0.81	0.8	0.84	*P* < 0.001
ICU Admission	No	221,330	92.29	18,499	7.71	Ref			
	Yes	39,990	62.43	24,068	37.57	7.49	7.32	7.67	*P* < 0.001
Hospital Type	Private	3,607	88	492	12	Ref			
	charity	690	83.43	137	16.57	1.73	1.37	2.19	*P* < 0.001
	Government universities hospitals	254,024	85.93	41,584	14.07	2.08	1.87	2.31	*P* < 0.001
	Other government hospitals	1,178	85.86	194	14.14	1.45	1.19	1.77	*P* < 0.001
	Non-governmental public	1,823	91.92	160	8.08	0.98	0.8	1.2	*P* < 0.001
Month of admission	2020-February	705	85.56	119	14.44	Ref			
	2020-March	16,283	86.17	2613	13.83	0.46	0.66	0.32	*P* < 0.001
	2020-April	15,490	86.99	2317	13.01	0.33	0.48	0.23	*P* < 0.001
	2020-May	13,138	88.49	1709	11.51	0.25	0.37	0.18	*P* < 0.001
	2020-June	17,229	88.22	2300	11.78	0.31	0.44	0.21	*P* < 0.001
	2020-July	25,414	86.13	4093	13.87	0.39	0.57	0.27	*P* < 0.001
	2020-August	20,025	85.66	3351	14.34	0.39	0.56	0.27	*P* < 0.001
	2020-September	19,300	86.9	2909	13.1	0.33	0.48	0.23	*P* < 0.001
	2020-October	29,168	84.47	5361	15.53	0.42	0.6	0.29	*P* < 0.001
	2020-November	39,523	83.64	7728	16.36	0.49	0.7	0.34	*P* < 0.001
	2020-December	23,902	84.39	4420	15.61	0.37	0.53	0.26	*P* < 0.001
	2021-January	15,390	86.51	2400	13.49	0.3	0.44	0.21	*P* < 0.001
	2021-February	14,763	88.74	1874	11.26	0.26	0.37	0.18	*P* < 0.001
	2021-March	10,990	88.89	1373	11.11	0.24	0.34	0.16	*P* < 0.001

^a^Confidence Interval

### Data collection

In this study, codes U07.1 and U07.2, defined as the international classification of diseases (ICD) codes in the 2019 version [34], were considered the basis for identifying patients with COVID-19 in hospitals. U07.1 is used when an individual’s infection with COVID-19 is based on confirmed diagnostic tests, regardless of the disease severity or clinical signs and symptoms. U07.2 is used when COVID-19 is clinically or epidemiologically diagnosed, but the results of laboratory tests are not conclusive, or these tests are not available. The required data for the present study was extracted from the inpatient document processing system [35] of the IHIO. This system was launched in 2019, aiming at accepting, processing, and handling hospital documents and records in IHIO, and is currently established in 1080 hospitals across the country. By enjoying the inpatient document processing system, all patient registered services can be viewed and handled electronically in the insurance organization. This system receives information from the hospital information systems (HIS) and, after the necessary insurance and service cost checks and validations, provides feedback results to the HIS system [36]. In the present study, the information from all hospitalized patients covered by IHIO identified by the final diagnosis of COVID-19 (codes U07.1 and U07.2) was extracted from the mentioned system at the onset of the COVID-19 pandemic until March 20, 2021. The variables including discharge time, geographical characteristics, provincial distribution, the patients’ status at discharge, and hospitalization duration in general and special wards, and etc. were selected from among the variables extracted from the system and were used in final analyses to evaluate the temporal, geographical, and age-gender distribution of patients and to estimate the odds of death due to COVID-19 in each of the above groups.

### Data analysis

The provincial distribution map of the total number of hospitalized cases, the number of patients hospitalized per 100,000 insured population, the percentage of ICU-admitted patients, the total number of ICU beds per day, the total number of beds per day of hospitalized cases insured by IHIO, the average hospitalization duration of patients, the total number of deaths, and the CFR of hospitalized patients were also plotted using GeoDA software. The multiple logistic regression model was used to investigate and identify the variables related to the odds of deaths in hospitalized patients by controlling for the confounding factors and also estimating the related odds ratio and 95% confidence intervals using STATA 14 software.

## Results

**Fig. 1:**
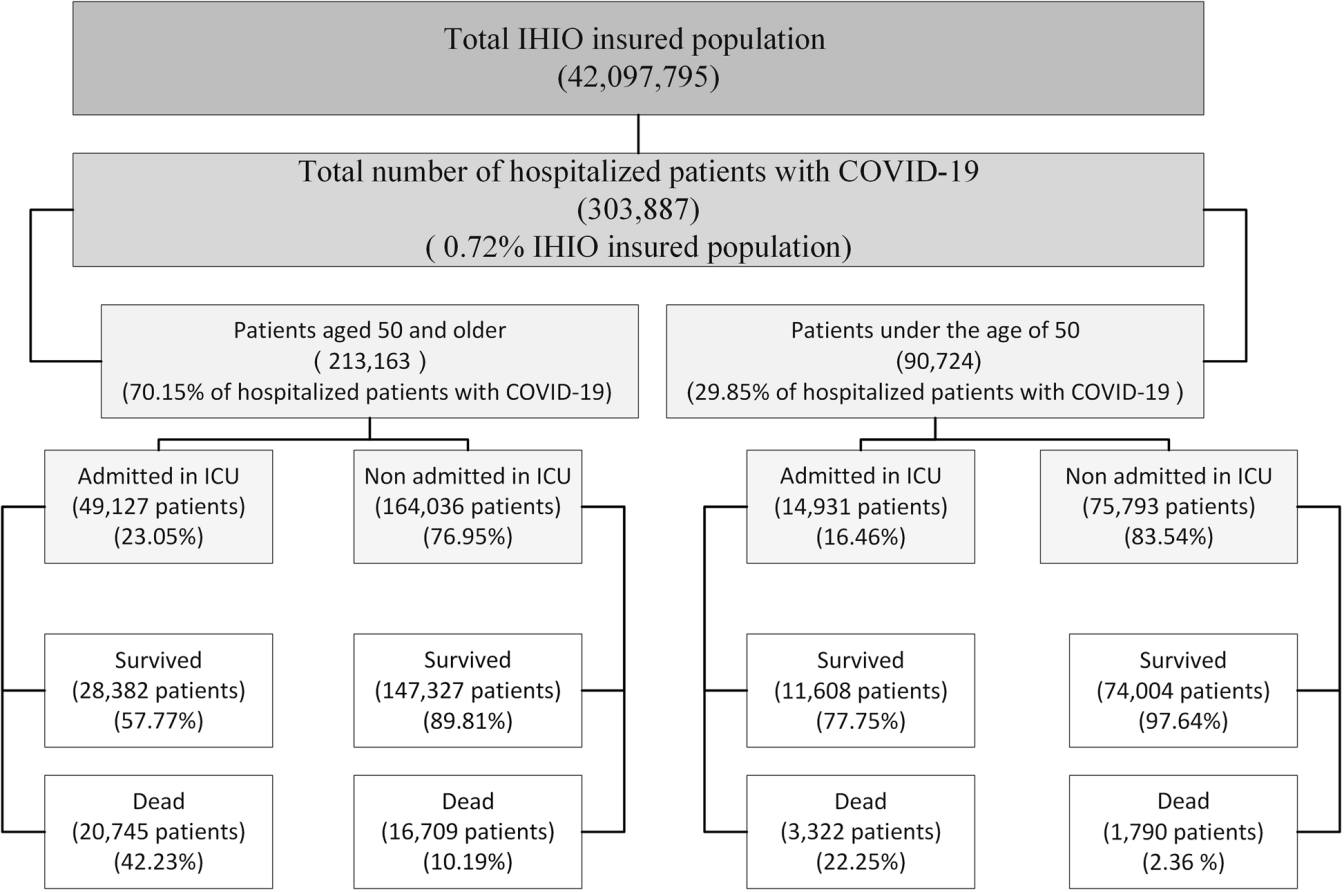
Hospitalization and death due to COVID-19 in the population insured by Iran Health Insurance Organization until March 20, 2021

During the first 14 months of the COVID-19 outbreak in Iran, 303,887 (0.72%) of the population insured by IHIO were hospitalized across the country due to this disease. The CFR from COVID-19 has been considerably different in various groups, from about 2.4% in patients under 50 years without ICU admission to 42.2% in patients over 50 years receiving ICU care (Fig. 1).

**Fig. 2:**
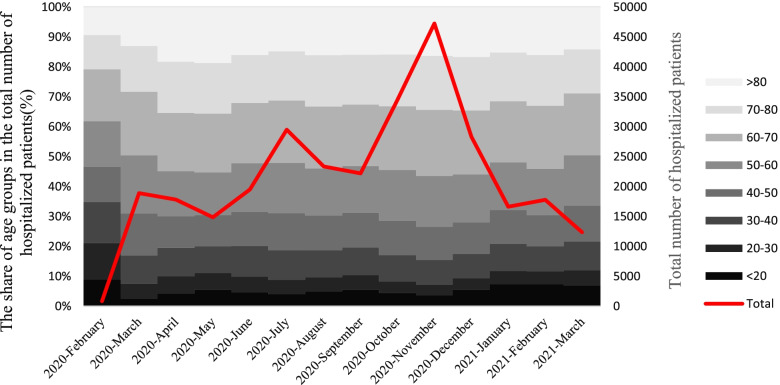
The time trend of the number of hospitalized patients with COVID-19 and the share of different age groups in the total number of COVID-19 hospitalized patients among the population insured by Iran Health Insurance Organization until March 20, 2021

During the studied time interval, three surge cases were observed in the number of hospitalized individuals, the highest of which was related to November 2020, so that the number of hospitalized patients increased from 34,529 in October to 47,251. The smoothing of this peak lasted until January. During this period, the age composition of hospitalized cases did not change significantly (Fig. 2).

**Fig. 3:**
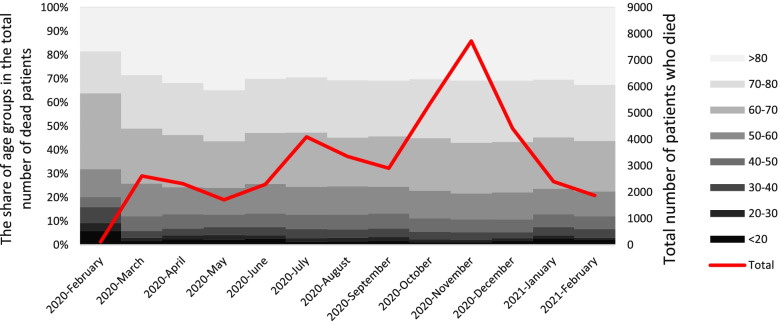
Time trend of the number of deaths due to COVID-19 and the share of different age groups in the total number of COVID-19 deaths among the population insured by Iran Health Insurance Organization until March 20, 2021

Following the November surge in the number of hospitalized cases, the number of deaths due to COVID-19 also increased remarkably from 5,361 cases in October to 7,728 cases in November. As can be seen, about 50% of deaths have occurred in the age group over 70 years during the entire time interval studied (Fig. 3).

**Fig. 4:**
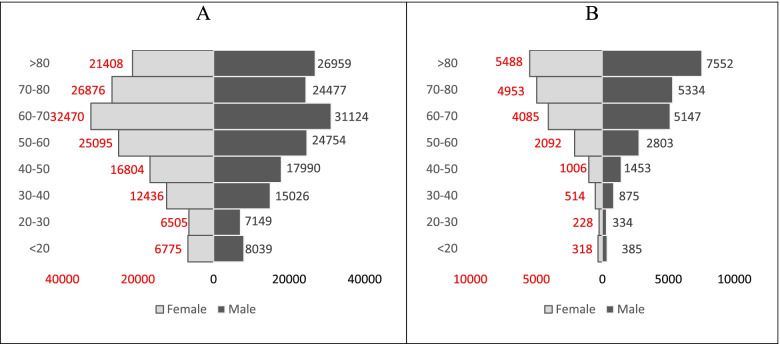
The population age pyramid: A- Total hospitalized patients, B- Patients died due to COVID-19 among the population insured by Iran Health Insurance Organization until March 20, 2021

In both gender groups, 60–70-year-old individuals had the highest number of hospitalizations due to this disease, while the CFR in the age group over 80 years was higher than that in other age groups (Fig. 4).

**Fig. 5:**
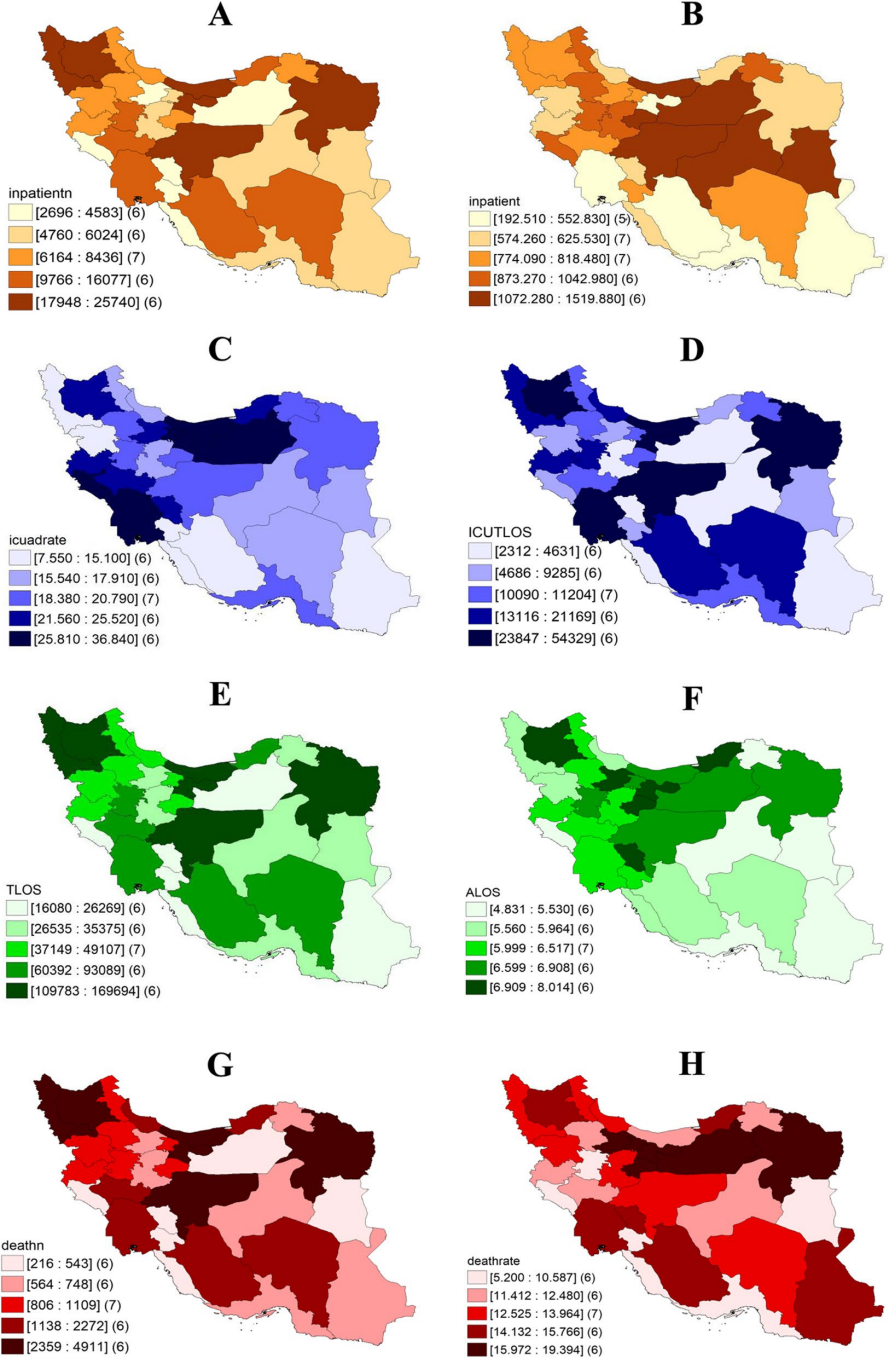
The provincial distribution of hospitalized patients due to the COVID-19 among the population insured by Iran Health Insurance Organization until March 20, 2021. A: Total number of hospitalized cases, B: Number of hospitalized patients per 100,000 insured population, C: Percentage of patients ICU-admitted, D: Total number of ICU-occupied bed-days, E: Total number of bed-days for hospitalized patients, F: Patients’ average length of stay, G: Total number of deaths, H: Percentage of death of hospitalized patients

Figure 5 shows the geographical distribution of important variables among the country’s provinces. As shown, Tehran, Isfahan, Mazandaran, Razavi Khorasan, East Azerbaijan, and West Azerbaijan provinces had the highest number of hospitalized cases among the population insured by IHIO. After adjusting for population covered by the organization in each province and examining the distribution of the number of hospitalized cases per 100,000 insured people, Mazandaran, Semnan, Qom, Yazd, Isfahan, and South Khorasan provinces were in the upper five ranks. ICU- admission rate of hospitalized patients varied from about 7% to 36%. In the six provinces of Khuzestan, Tehran, Ilam, Mazandaran, Alborz, and Semnan, the hospitalization percentage in ICU was over 25% in average (Fig. 5).

Total number of ICU-occupied bed-days varied between 2,312 to 54,329 days. Tehran, Mazandaran, Razavi Khorasan, East Azerbaijan, Khuzestan, and Isfahan provinces were located in the upper five ranks. The Total number of bed-days for hospitalized patients (sum of critical and non-critical beds) ranged from 16,060 days to 169,694 days. The highest total number of beds-days belonged to Tehran, Mazandaran, East Azerbaijan, West Azerbaijan, Isfahan, and Razavi Khorasan provinces. The average hospitalization length of stay varied from 4.8 days to about 8 days, and in this regard, ChaharM & Bakhtiari, Golestan, East Azerbaijan, Qazvin, Tehran, and Qom provinces were in the last five ranks. According to studies, the number of deaths varied from 216 to 4,911 cases. Tehran, Mazandaran, Isfahan, Razavi Khorasan, East Azerbaijan, and West Azerbaijan provinces had the highest total number of deaths, but in terms of the percentage of deaths of hospitalized patients, Razavi Khorasan, Semnan, Qom, Tehran, Alborz, and Qazvin provinces were in the last five ranks (Fig. 5). (For underlying data of this figure see table 3 in additional file 3).

The findings of multiple logistic regression analysis regarding the factors related to the death of hospitalized patients due to COVID-19 showed that the odds of death due to COVID-19 in the insured population of Foreign Citizen Fund was higher than that of the insured population of other funds (OR = 1.45, CI = 95% [1.28–1.66]). Age was a significant factor affecting the patients’ death, so that the odds ratio of death in all age groups was higher than one and significant compared to that of the reference age group (less than 20 years old). The odds of death in the age group over 80 years were 9.6 times higher than that in the under 20-year age group (OR = 9.65, CI = 95% [8.89–10.49]). The odds of death in females were significantly lower than in males (OR = 0.81, CI = 95% [0.80–0.84]). Patients admitted to the ICU had nearly 7.5 times more odds of death than those not admitted to the ICU (OR = 7.49, CI = 95% [7.32–7.67]) (Table 1).

Compared to the reference province, which was East Azerbaijan province, the odds ratio is evident in both protection and vulnerability. For example, this ratio was about 2 in Sistan and Baluchestan province (OR = 2.01, CI 95% [1.83–2.22]) and in other provinces such as Kohgiluyeh and Boyer Ahmad (OR = 0.54, CI 95% [0.47–0.63] And Ilam (OR = 0.54, 95% CI [0.488–0.62]) was about 0.5. (see Table A2 in additional file 2).

## Discussion

The present study was conducted to investigate the different consequences of patients hospitalized due to COVID-19 in the population insured by IHIO. Based on the results of this study, the number of hospitalization cases and rates in the target population were 303,887 and 0.72%, respectively. According to the Ministry of Health statistics, in this period, the total number of hospitalizations across the country was about 404,000 [37], which given the population of the country (about 84 million people) [38], the hospitalization rate in the whole country has been 0.52%. In the hospitalized population covered by the IHIO, 42,567 patients hospitalized due to COVID-19 died (14% death rate). In Khoshnood et al.’s study, the CFR among 1,083 patients hospitalized in Hazrat Rasoul Akram Hospital in Tehran was reported to be 10.8% [39]. In Zali et al.’s study on about 16,000 patients hospitalized in the hospitals of Shahid Beheshti University of Medical Sciences, the total CFR was reported to be 10.05% [40]. In Bellan et al.’s study, the CFR among patients hospitalized due to COVID-19 was estimated at 29.7% [41]. In Navaratnam’s study, the rate was reported to be 30.8% [42]. In a multinational cohort study by Bertsimas et al., the inter-hospital CFR was reported to be 26.84% [43]. Asch et al. also reported the inter-hospital CFR in the United States between 9.06% and 15.65% [44]. There are various reasons for these differences, including differences in disease severity, differences in demographic-epidemiological characteristics of the study population, healthcare infrastructure, and behavioral-cultural factors of the population. Numerous studies have been conducted to investigate the causes of different CFRs due to COVID-19. For example, based on the results of Liang et al.’s study, the CFR due to COVID-19 among countries was positively correlated with population aged 65 or older and the transport infrastructure quality. Furthermore, it negatively associated with the number of diagnostic tests performed [45]. In Annakan Vnavaratnam et al.’s study, aging, male gender, higher deprivation, mixed ethnicity, and many comorbid diseases, including moderate to severe liver diseases, were associated with the inter-hospital odds of death due to COVID-19 [42]. According to the results of Zali et al.’s study, masculinity (Hazard Ratio = 1.19), being over 65 years old (Hazard Ratio = 2.18), and ICU admission (Hazard Ratio = 3.93) were significant risk factors related to the survival duration of patients who died due to COVID-19 [40].

Based on the results of the present study, a set of factors, age, gender, geographical location, disease severity (ICU admission has been considered a criterion of disease severity), date of infection, type of insurance fund, and hospitalization center ownership have affected patients’ odds of death to various degrees. The odds of death due to COVID-19 were significantly lower in women than in men. Other studies on COVID-19 have mentioned gender-based differences in fatality due to COVID-19 and better outcomes in women [46–48]. Ahrenfeldt et al.’s showed that deaths due to COVID-19 were higher in men than in women in all age groups and all regions of Europe [49]. According to Raza et al.’s [47] study, facing COVID-19, women had lower rates of infection and hospitalization, better prognosis, and lower CFR compared to men. This inequality may be explained by various mechanisms, including differences in innate and acquired immune responses, genetic factors, interactions between sex hormones and immune factors, as well as gender-specific behavioral differences [46, 47]. Some studies conducted in Iran have also reported such gender differences in the consequences of COVID-19 [39, 50]. Waris has attributed these differences to biological, social, occupational, religious, psychological, cultural, and lifestyle factors [51]. Such inequalities was reported in the epidemic of other infectious diseases such as SARS and MERS [16, 52].

Findings show that odds of death in the age group over 80 years was about 9.6 times higher than that of individuals under 20 years. As shown in Fig. 1, a significant percentage of hospitalized patients (70.15%) were over 50 years old. This group of people has experienced a proportionately higher percentage of ICU hospitalizations than othwe (23.05% vs. 16.48%). For all health conditions, the availability of reliable data by age is crucial for epidemiological analyses and also for monitoring the relative priority of different age groups in providing policy responses. In all countries, the odds of death due to COVID-19 increases significantly with age. Therefore, the elderly account for the majority of COVID-19 deaths. Even in countries where population aging is limited [53]. Zali et al.’s study indicated that the highest number of cases of infection with COVID-19 belonged to the 25–64-year-old age group, while the highest number of CFRs occurred in the age group over 64 years [40]. In Ebrahimi et al.’s study, 71% of all deaths were in the age group over 60 years and the odds of death in the age group over 60 years were 5 times higher than others [54]. According to the results of Biswas et al.’s study, the odds of death for individuals aged 50 years and over are 15.4 times higher than that of individuals under 50 years old [48]. As shown in Figs. 2 and 3, the age composition of both the hospitalized and deceased populations due to COVID-19 did not change significantly during the study period. One of the reasons for the significant increase in the odds of death at older ages could be the higher prevalence of underlying diseases in these age groups. The associations of several comorbid diseases with the severity and CFR related to COVID-19 have been demonstrated. However, significant differences in estimating the prevalence of comorbid diseases and their effects on COVID-19 consequences and CFR are evident in studies [55]. Based on Thakur et al.’s study, the most common comorbidities include high blood pressure, obesity, diabetes, cardiovascular diseases, and kidney diseases. Patients with cerebrovascular accidents and cardiovascular diseases have had higher disease severity and CFRs facing COVID-19 [55]. One of the limitations of this study is that it was not possible to distinguish the effect of age from the underlying diseases due to data restrictions. However, due to the high dependence of the prevalence of comorbidities on aging, it seems that an individual’s age can be an appropriate proxy for the prevalence of comorbidity. For example, according to the global burden of disease study 2019, 79.36% of cardiovascular diseases, 52.44% of chronic respiratory diseases, 61.49% of diabetes, and 53.47% of neoplasms are globally prevalent in the age group over 50 years [56], while they are 22.95% of the world’s population [57].

Another factor affecting the odds of death due to COVID-19 is the type of insurance fund. According to the findings, the odds of death of the foreign (Including refugees and migrants (was higher than other insured individuals. Refugees are a substantial population in terms of disease control [58]. Therefore, effective health monitoring and data collection are essential for understanding the health needs of immigrants and refugees, assessing the capacity of the health system, and prioritizing to ensure that refugee and immigrant care is integrated into the overall healthcare system [59]. According to the United Nations High Commissioner for Refugees (UNHCR), Iran is one of the 10 countries hosting the largest number of refugees globally [58]. According to statistical reports, most immigrants and refugees living in Iran are Afghans [60]. These individuals face many challenges, having been intensified during the COVID-19 period. Only about 124,000 (6%) Afghans in Iran have registered for health insurance, so a wide range of these individuals still lack insurance coverage, leading to reduce their willingness to be hospitalized due to high medical costs. Given that most of the refugees in Iran are daily-paid, they have faced decreased income due to quarantine conditions, and consequently, their visit rates to the clinics have decreased due to lack of financial access [58]. The results of Hayward et al.’s study show the disproportionate distribution of COVID-19 deaths as well as increased CFR due to all causes among immigrants in high-income countries in 2020.

The findings showed that individuals hospitalized in Government universities hospitals have had higher odds of death. One of the reasons for this can be the high burden of hospitalization of patients in these centers. The total number of cases hospitalized in private hospitals has been 3,607 versus 254,024 in Government universities hospitals. According to Olivas-Martínez et al.’s study, 45% of patients who did not survive were indicated for admission to the ICU but did not receive the necessary care due to the lack of providing ICU beds, intensive care, and forced intermittent ventilation. Therefore, overcrowding has been one of the causes of COVID-19 deaths in medical centers. Another reason could be the better socio-economic status of patients hospitalized in private centers. The costs for private-sector services are significantly higher than those for public-sector services. For example, in 2020, the cost of one day of ICU accommodation in private hospitals was about 24 million rials, while in public centers, this amount was about 9.5 million rials [61]. Therefore, low-income individuals cannot usually afford to use the services of private treatment centers.

According to the results, the odds of death for the ICU-admitted patients was 7.5 times higher than for others, and 37.75% of these patients have died. Zali et al.’s study show that the odds of death of ICU admission have been three times higher than that of others [40]. Given the results of Grasselli et al.’s study, the death rate of ICU-admitted patients was 48.7% [62]. According to Armstrong et al.’s research, the death rate within the ICU in COVID-19 patients ranged from zero to 84.6% [63]. The death rate within the ICU was significantly higher than other viral types of pneumonia. The reason can be attributed to both the disease process itself and the difficulty in providing reliable ICU services during the pandemic [63].

The findings showed that the hospitalization location of an individual were influential factors in the odds of death for patients with COVID-19 so that the odds of death were higher in 14 provinces compared to the reference province (East Azerbaijan). This rate varied from 0.54 in Ilam and Kohgiluyeh and Boyer-Ahmad to 1.66 in Razavi Khorasan and 2.01 in Sistan and Baluchestan. Across regions disparities were evident in death due to COVID-19 and other measures such as hospitalization rate, length of stay and admission rate to the ICU. Such geographical differences have also been evident in other domestic studies as well as studies conducted in other countries of the world [64, 65], which can be attributed to the level of development of regions, climatic characteristics, cultural differences, differences in the distribution of disease burden, and differences in providing healthcare infrastructures or service delivery processes. For example, according to Gupta et al.’s study, relatively hot and dry regions at altitudes lower than India were more susceptible to infection with COVID-19 transmission [64].

At the time interval of about one year, Mazandaran, Tehran, East Azerbaijan, West Azerbaijan, Razavi Khorasan, and Isfahan provinces were in the upper five provinces in terms of hospitalization rate for patients with COVID-19 in the present study. However, after adjusting the insured population of each province, although Mazandaran and Isfahan provinces were still at the top, the pattern was different for other provinces, so that Tehran province was among the top five ranks. The status of the indicator in East Azerbaijan, West Azerbaijan, and Razavi Khorasan provinces improved. In terms of hospitalization rate per 100,000 insured population, Mazandaran, Isfahan, South Khorasan, Yazd, Semnan, and Qom provinces were in the upper five ranks. Other studies have been conducted on the geographical/spatial distribution of COVID-19 in Iran [24, 66, 67]. Due to differences in study time and population studied, it is not possible to completely compare the results of the studies. For example, in the study of Khalagi et al. Ardabil, Golestan and Khuzestan provinces had the highest prevalence and Alborz, Hormozgan and Kerman provinces had the lowest [67].

It was observed that in some provinces the chance of death due to COVID-19 was significantly higher than others, including Sistan & Baluchestan (OR = 2.01), Razavi Khorasan (OR = 1.66), Qom (OR = 1.31), Gilan (OR = 1.31), Golestan (OR = 1.26) and Fars ( OR = 1.26). Various studies indicate disparities in various fields including socio-economic development [68, 69], demographic characteristics [70], epidemiology of diseases [71], health resources [72] and health outcomes [73, 74] across provinces that each of them can affect the prevalence of COVID-19 and its consequences. For example, Mirfallah Nasiri's study showed that Tehran, Isfahan, East Azerbaijan, Gilan and Mazandaran provinces are among the province with the highest aging rates [70]. According to the Statistics Center of Iran, the provinces of Tehran, Razavi Khorasan, Isfahan and Mazandaran had the highest volume of travel, so that in 2020, their total intra-provincial and extra-provincial trips were 1,137,000, 1,042,000, 828,000 and 828,000, respectively [75]. The high volume of trips can be one of the causes of high prevalence and mortality in the above provinces. Making a sound judgment about the causes of differences between provinces requires further study and evaluation of the impact of each of the underlying factors.

health system performance in Iran has been presented, each of which can play an important role in explaining There are inter-provincial differences in hospitalization, length of hospital stays, admission to the ICU, length of stay in the ICU, and chance of death due to Covid 19.

One of the important limitations of this study is the impossibility of accessing the patient’s clinical information such as underlying diseases, patient’s clinical status, and details of treatment interventions. An attempt was made to remove this limitation by using multiple proxies. For example, aging has been considered a proxy for underlying diseases, and receiving ICU services has been considered a proxy for the disease severity.

## Conclusion

Men, the elderly, vulnerable target groups covered by IHIO such as foreign, ICU-admitted individuals, and individuals hospitalized in public university hospitals have had higher odds of death due to COVID-19 than others. On the other hand, the odds of death have been disproportionately varied in different geographical areas. Understanding the factors affecting the CFR of this disease and the severity of the impressibility of different geographical areas from this crisis is one of the fundamental components of policy-making to deal with the pandemic and try to reduce its harmful effects.

## Supplementary Information

**Additional file 1: Table A1.** Total population, IHIO Insured population, hospital numbers and number of hospital beds in the provinces of Iran

**Additional file 2: Table A2.** Death of patients hospitalized due to COVID-19 in the population insured by Iran Health Insurance Organization until March 20, 2021, by province (based on the multiple logistic regression model).

**Additional file 3: Table A3.** Detailed data of Figure 5 (The provincial distribution of hospitalized patients due to the COVID-19 among the population insured by Iran Health Insurance Organization until March 20, 2021).

## Data Availability

The datasets used and/or analyzed during the current study are available from the corresponding author on reasonable request.

## Abbreviations

IHIO: Iran Health Insurance Organization
OR: Odds Ratio
ICU: Intensive Care Unit
CFR: Case Fatality Rate
WHO: World Health Organization
DALY: Disability Adjuster Life Years
MoHME: Ministry of Health and Medical Education
HIS: Hospital information systems
CI: Confidence Interval
