# Supplementary material for: National geographical pattern of COVID-19 hospitalization, case fatalities, and associated factors in patients covered by Iran Health Insurance Organization
Source: BMC Public Health. 2022 Jun 30;22:1274. doi: 10.1186/s12889-022-13649-0 (PMC9243909; doi:10.1186/s12889-022-13649-0)
Supplement: Supplementary file 1 — Additional file 1: Table A1. Total population, IHIO Insured population, hospital numbers and number of hospital beds in the provinces of Iran [file 12889_2022_13649_MOESM1_ESM.docx]

**Table A1.** Total population, IHIO Insured population, hospital numbers and number of hospital beds in the provinces of Iran

| **Province name** | **Total Population** | **IHIO Insured population** | **Share of IHIO Insured population of the total population of the country (%)** | **Number of hospitals** | **Number of hospital beds** | **Number of ICU beds** |
| --- | --- | --- | --- | --- | --- | --- |
| Eeast Azerbaijan | 4,051,000 | 2,228,021 | 55.00 | 46 | 7,719 | 566 |
| West Azerbaijan | 3,439,000 | 2,369,369 | 68.90 | 37 | 4,995 | 446 |
| Ardabil | 1,306,000 | 856,438 | 65.58 | 18 | 2,716 | 145 |
| Isfahan | 5,343,000 | 1,749,655 | 32.75 | 69 | 9,114 | 669 |
| Alborz | 2,913,000 | 748,179 | 25.68 | 18 | 3,009 | 329 |
| Ilam | 602,000 | 392,762 | 65.24 | 12 | 893 | 93 |
| Bushehr | 1,251,000 | 458,431 | 36.65 | 19 | 1,438 | 105 |
| Tehran | 13,973,000 | 3,808,235 | 27.25 | 180 | 28,506 | 3,093 |
| ChaharM & Bakhtiari | 988,000 | 631,670 | 63.93 | 12 | 1,804 | 100 |
| South Khorasan | 822,000 | 513,666 | 62.49 | 17 | 1,398 | 126 |
| Razavi Khorasan | 6,871,000 | 4,209,027 | 61.26 | 68 | 10,291 | 821 |
| North Khorasan | 899,000 | 706,026 | 78.53 | 12 | 1,404 | 95 |
| Khuzestan | 4,936,000 | 2,570,575 | 52.08 | 56 | 8,980 | 725 |
| Zanjan | 1,107,000 | 623,791 | 56.35 | 14 | 2,092 | 135 |
| Semnan | 764,000 | 237,842 | 31.13 | 12 | 1,740 | 163 |
| Sistan & Baluchestan | 3,045,000 | 2,472,657 | 81.20 | 22 | 3,065 | 165 |
| Fars | 5,051,000 | 3,032,105 | 60.03 | 77 | 9,874 | 661 |
| Qazvin | 1,336,000 | 559,939 | 41.91 | 16 | 2,146 | 169 |
| Qom | 1,398,000 | 574,849 | 41.12 | 10 | 2,262 | 145 |
| Kurdistan | 1,675,000 | 1,163,583 | 69.47 | 20 | 2,952 | 147 |
| Kerman | 3,341,000 | 1,728,613 | 51.74 | 42 | 5,662 | 379 |
| Kermanshah | 1,999,000 | 1,307,856 | 65.43 | 25 | 3,327 | 287 |
| Kohgiluyeh & BoyerA | 753,000 | 514,352 | 68.31 | 11 | 1,671 | 78 |
| Golestan | 1,975,000 | 1,277,696 | 64.69 | 25 | 2,995 | 202 |
| Gilan | 2,571,000 | 1,438,465 | 55.95 | 36 | 3,822 | 245 |
| Lorestan | 1,801,000 | 1,244,418 | 69.10 | 27 | 2,486 | 226 |
| Mazandaran | 3,391,000 | 1,630,546 | 48.08 | 47 | 5,690 | 568 |
| Markazi | 1,478,000 | 608,727 | 41.19 | 21 | 2,266 | 179 |
| Hormozgan | 1,942,000 | 1,113,267 | 57.33 | 22 | 2,573 | 187 |
| Hamadan | 1,779,000 | 1,189,197 | 66.85 | 21 | 3,064 | 243 |
| Yazd | 1,236,000 | 318,380 | 25.76 | 20 | 2,814 | 184 |
| Iran (Islamic Republic) | 84,036,000 | 42,278,338 | 50.31 | 1,032 | 142,768 | 11,676 |
