# Supplementary material for: National geographical pattern of COVID-19 hospitalization, case fatalities, and associated factors in patients covered by Iran Health Insurance Organization
Source: BMC Public Health. 2022 Jun 30;22:1274. doi: 10.1186/s12889-022-13649-0 (PMC9243909; doi:10.1186/s12889-022-13649-0)
Supplement: Supplementary file 2 — Additional file 2: Table A2. Death of patients hospitalized due to COVID-19 in the population insured by Iran Health Insurance Organization until March 20, 2021, by province (based on the multiple logistic regression model). [file 12889_2022_13649_MOESM2_ESM.docx]

**Table A2.** Death of patients hospitalized due to COVID-19 in the population insured by Iran Health Insurance Organization until March 20, 2021, by province (based on the multiple logistic regression model)

| **Province** | **Survived** | | **Dead** | | **Odds Ratio** | | | **P-Value** |
| --- | --- | --- | --- | --- | --- | --- | --- | --- |
|  | **Frequency** | **%** | **Frequency** | **%** | **OR** | **95% Confidence Interval** | |  |
| Eeast Azerbaijan | 15,286 | 85.17 | 2,662 | 14.83 | Ref |  |  |  |
| West Azerbaijan | 16,342 | 87.39 | 2,359 | 12.61 | 1.12 | 1.06 | 1.2 | P<0.001 |
| Ardabil | 6,528 | 87.28 | 951 | 12.72 | 1.04 | 0.95 | 1.14 | 0.36 |
| Isfahan | 18,612 | 86.23 | 2,972 | 13.77 | 0.88 | 0.83 | 0.95 | P<0.001 |
| Alborz | 4,915 | 81.59 | 1,109 | 18.41 | 1.2 | 1.14 | 1.36 | P<0.001 |
| Ilam | 3,217 | 89.86 | 363 | 10.14 | 0.54 | 0.48 | 0.62 | P<0.001 |
| Bushehr | 2,443 | 90.24 | 263 | 9.76 | 0.9 | 0.78 | 1.05 | 0.17 |
| Tehran | 16,093 | 80.61 | 3,827 | 19.39 | 1.07 | 1.01 | 1.14 | 0.01 |
| ChaharM & Bakhtiari | 3,231 | 85.82 | 534 | 14.18 | 0.95 | 0.85 | 1.07 | 0.41 |
| South Khorasan | 5,098 | 90.37 | 543 | 9.63 | 0.66 | 0.59 | 0.74 | P<0.001 |
| Razavi Khorasan | 20,829 | 80.92 | 4,911 | 19.08 | 1.66 | 1.58 | 1.77 | P<0.001 |
| North Khorasan | 5,667 | 88.59 | 730 | 11.41 | 0.86 | 0.78 | 0.95 | P<0.001 |
| Khuzestan | 12,119 | 85.28 | 2,092 | 14.72 | 1.16 | 1.08 | 1.25 | P<0.001 |
| Zanjan | 5,700 | 87.61 | 806 | 12.39 | 0.87 | 0.79 | 0.96 | P<0.001 |
| Semnan | 2,445 | 83.48 | 484 | 16.52 | 0.83 | 0.74 | 0.94 | P<0.001 |
| Sistan & Baluchestan | 4,012 | 84.29 | 748 | 15.71 | 2.01 | 1.83 | 2.22 | P<0.001 |
| Fars | 13,805 | 85.87 | 2,272 | 14.13 | 1.26 | 1.18 | 1.35 | P<0.001 |
| Qazvin | 3,851 | 84.03 | 732 | 15.97 | 1.08 | 0.99 | 1.2 | 0.09 |
| Kurdistan | 5,844 | 87.46 | 838 | 12.54 | 0.95 | 0.87 | 1.04 | 0.3 |
| Qom | 5,059 | 82.07 | 1,105 | 17.93 | 1.31 | 1.21 | 1.43 | P<0.001 |
| Kerman | 11,705 | 87.84 | 1,676 | 12.53 | 1.1 | 1.03 | 1.19 | P<0.001 |
| Kermanshah | 7,160 | 87.52 | 1,021 | 12.48 | 0.85 | 0.79 | 0.93 | P<0.001 |
| Kohgiluyeh & BoyerA | 3,938 | 94.8 | 216 | 5.2 | 0.54 | 0.47 | 0.63 | P<0.001 |
| Golestan | 8,431 | 84.23 | 1,578 | 15.77 | 1.28 | 1.19 | 1.38 | P<0.001 |
| Gilan | 7,258 | 86.04 | 1,178 | 13.96 | 1.31 | 1.21 | 1.43 | P<0.001 |
| Lorestan | 8,628 | 88.35 | 1,138 | 11.65 | 0.76 | 0.7 | 0.83 | P<0.001 |
| Mazandaran | 18,426 | 88.46 | 2,404 | 11.54 | 0.68 | 0.64 | 0.73 | P<0.001 |
| Markazi | 4,985 | 87.17 | 734 | 12.83 | 0.85 | 0.78 | 0.94 | P<0.001 |
| Hormozgan | 5,160 | 89.41 | 611 | 10.59 | 1.01 | 0.92 | 1.13 | 0.77 |
| Hamadan | 10,268 | 90.32 | 1,101 | 9.68 | 0.63 | 0.59 | 0.69 | P<0.001 |
| Yazd | 4,275 | 88.34 | 564 | 11.66 | 0.78 | 0.7 | 0.87 | P<0.001 |
