# Supplementary material for: National geographical pattern of COVID-19 hospitalization, case fatalities, and associated factors in patients covered by Iran Health Insurance Organization
Source: BMC Public Health. 2022 Jun 30;22:1274. doi: 10.1186/s12889-022-13649-0 (PMC9243909; doi:10.1186/s12889-022-13649-0)
Supplement: Supplementary file 3 — Additional file 3: Table A3. Detailed data of Figure 5 (The provincial distribution of hospitalized patients due to the COVID-19 among the population insured by Iran Health Insurance Organization until March 20, 2021). [file 12889_2022_13649_MOESM3_ESM.docx]

**Table A3.** Detailed data of Figure 5 (The provincial distribution of hospitalized patients due to the COVID-19 among the population insured by Iran Health Insurance Organization until March 20, 2021)

| Province name | Total number of hospitalized cases | Number of hospitalized patients per 100,000 insured population | Percentage of patients ICU-admitted | Total number of ICU-occupied bed-days | Total number of bed-days for hospitalized patients | Patients’ average length of stay | Total number of deaths | Percentage of death of hospitalized patients |
| --- | --- | --- | --- | --- | --- | --- | --- | --- |
| Eeast Azerbaijan | 17948 | 805.56 | 23.23 | 30,071 | 123,423 | 6.93 | 2,662 | 14.83 |
| West Azerbaijan | 18701 | 789.28 | 14.38 | 15,105 | 109,783 | 5.87 | 2,359 | 12.61 |
| Ardabil | 7479 | 873.27 | 16.21 | 10,373 | 45,973 | 6.15 | 951 | 12.72 |
| Isfahan | 21584 | 1233.61 | 20.35 | 30,990 | 146,688 | 6.80 | 2,972 | 13.77 |
| Alborz | 6024 | 805.15 | 25.81 | 11,204 | 41,592 | 6.91 | 1,109 | 18.41 |
| Ilam | 3580 | 911.49 | 28.10 | 4,686 | 19,055 | 5.32 | 363 | 10.14 |
| Bushehr | 2696 | 588.09 | 14.58 | 2,993 | 16,080 | 5.96 | 263 | 9.76 |
| Tehran | 19965 | 524.26 | 36.85 | 54,329 | 159,309 | 5.01 | 3,872 | 19.39 |
| ChaharM & Bakhtiari | 3765 | 596.04 | 21.57 | 2,332 | 26,013 | 6.90 | 534 | 14.18 |
| South Khorasan | 5641 | 1098.19 | 15.55 | 6,310 | 27,230 | 4.83 | 543 | 9.63 |
| Razavi Khorasan | 25740 | 611.54 | 19.18 | 35,033 | 169,694 | 6.60 | 4,911 | 19.08 |
| North Khorasan | 6397 | 906.06 | 20.79 | 10,254 | 35,375 | 5.53 | 730 | 11.41 |
| Khuzestan | 14211 | 552.83 | 26.56 | 23,847 | 90,283 | 6.35 | 2,092 | 14.72 |
| Zanjan | 6506 | 1042.98 | 18.38 | 10,090 | 42,402 | 6.52 | 806 | 12.39 |
| Semnan | 2929 | 1231.49 | 31.21 | 2,312 | 20,061 | 6.85 | 484 | 16.52 |
| Sistan & Baluchestan | 4760 | 192.51 | 13.00 | 3,551 | 26,269 | 5.52 | 748 | 15.71 |
| Fars | 16077 | 530.23 | 15.11 | 20,391 | 93,089 | 5.79 | 2,272 | 14.13 |
| Qazvin | 4583 | 818.48 | 25.53 | 8,888 | 31,753 | 6.93 | 732 | 15.97 |
| Qom | 6164 | 1072.28 | 19.78 | 10,940 | 42,989 | 6.98 | 1,105 | 17.93 |
| Kurdistan | 6682 | 574.26 | 14.74 | 9,285 | 37,149 | 5.56 | 838 | 12.54 |
| Kerman | 13381 | 774.09 | 17.91 | 21,169 | 77,739 | 5.81 | 1,676 | 12.53 |
| Kermanshah | 8181 | 625.53 | 21.57 | 15,529 | 49,107 | 6.01 | 1,021 | 12.48 |
| Kohgiluyeh & BoyerA | 4154 | 807.62 | 7.56 | 5,002 | 25,514 | 6.14 | 216 | 5.20 |
| Golestan | 10009 | 783.36 | 22.70 | 9,239 | 69,531 | 6.95 | 1,578 | 15.77 |
| Gilan | 8436 | 586.46 | 15.72 | 14,064 | 48,282 | 5.73 | 1,178 | 13.96 |
| Lorestan | 9766 | 784.78 | 23.80 | 10,191 | 60,392 | 6.19 | 1,138 | 11.65 |
| Mazandaran | 20830 | 1277.49 | 26.61 | 27,774 | 139,134 | 6.68 | 2,404 | 11.54 |
| Markazi | 5719 | 939.50 | 17.01 | 4,063 | 34,307 | 6.00 | 734 | 12.83 |
| Hormozgan | 5771 | 518.38 | 18.77 | 10,828 | 28,279 | 4.90 | 611 | 10.59 |
| Hamadan | 11369 | 956.02 | 19.61 | 13,116 | 76,988 | 6.77 | 1,101 | 9.68 |
| Yazd | 4839 | 1519.88 | 16.39 | 4,631 | 26,535 | 5.48 | 564 | 11.66 |
| Iran (Islamic Republic) | 303887 | 718.78 | 21.08 | 435,590 | 1,940,018 | 6.13 | 42,567 | 14.01 |
